# Optic Nerve Sheath Diameter as a Prognostic Tool for Mortality in Intensive Care Unit Patients

**DOI:** 10.7759/cureus.100659

**Published:** 2026-01-03

**Authors:** Fahimuddin Syed, Mohamed F Hendi, Nayeem Ghouse Mohammed, Zeyad Faoor Medhat Alrais, Waseem Sheikh

**Affiliations:** 1 Intensive Care Unit, Rashid Hospital, Dubai Academic Health Corporation, Dubai, ARE; 2 Clinical Faculty Critical Care, Mohammed Bin Rashid University of Medicine and Health Sciences, Dubai, ARE; 3 Critical Care Medicine, Rashid Hospital, Dubai Academic Health Corporation, Dubai, ARE

**Keywords:** icu, intracranial pressure, mortality, optic nerve sheath diameter, prognostic tool

## Abstract

Background: The relationship between optic nerve sheath diameter (ONSD) and mortality outcomes in critically ill intensive care unit (ICU) patients is an area of increasing interest. This study aimed to explore whether ONSD can serve as a reliable predictor of mortality in ICU patients, potentially offering a non-invasive tool to guide clinical decision-making and patient management.

Materials and methods: This prospective cohort study included critically ill ICU patients aged 14 years and above, excluding those with pre-existing optic nerve disorders, traumatic brain injury (TBI), or intracerebral hemorrhage (ICH). Demographic and clinical data, including age, sex, comorbidities, injury severity score, Glasgow Coma Scale (GCS), and laboratory data, such as liver function tests (LFTs), hemoglobin (Hb) level, and procalcitonin (PCT) level, were collected. ONSD was measured on Day 1 and Day 10 using transorbital ultrasonography. Statistical analysis was performed using IBM SPSS Statistics for Windows, Version 21 (released 2012; IBM Corp., Armonk, New York, United States) to determine the association between ICU mortality and other outcomes.

Results: The patients in this study had a mean age of 58.29 years, with the majority presenting with comorbidities such as diabetes mellitus (DM) and hypertension (HTN). Clinical outcomes showed significant reductions in C-reactive protein (CRP) over ten days, with a notable prevalence of sepsis (91.2%) and a survival rate of 66.2%, while the ONSD values on ICU admission day were significantly higher in non-survivors (mean right/left ONSD = 4.02/3.97 mm) versus survivors (mean right/left ONSD = 3.68/3.67 mm), with p-values of 0.001 indicating statistical significance.

Conclusions: ONSD measurement is a valuable prognostic tool in ICU patients, correlating with mortality outcomes, especially in septic patients, and can help guide timely interventions for better outcomes.

## Introduction

Optic nerve sheath diameter (ONSD) measurement has emerged as a valuable tool for assessing intracranial pressure (ICP) and predicting mortality outcomes in intensive care unit (ICU) patients, particularly in those with traumatic brain injury (TBI). The optic nerve sheath, which surrounds the optic nerve, is highly sensitive to ICP changes. As ICP increases, the sheath expands, making ONSD an indirect but reliable indicator of increased ICP.

Studies have shown that increased ONSD is independently associated with higher ICU mortality rates in severe TBI patients [1,2]. For instance, an ONSD cutoff ≥ 7.3 mm demonstrated a sensitivity of 86.4% and specificity of 74.6% in predicting mortality [1]. This relationship has led to the use of ONSD measurement via ultrasonography as a tool for early detection of increased ICP in various clinical settings, particularly in the ICU [3].

In the ICU, patients are often at high risk of developing conditions that can lead to increased ICP, such as TBI, stroke, encephalopathy, sepsis, and intracranial hemorrhage [4]. ICP is a serious complication that can occur in various neurologic injuries and poses a major challenge in ICUs [5].

Elevated ICP is associated with poor outcomes, including brain damage, disability, and increased mortality. Timely identification of elevated ICP can significantly impact patient prognosis by guiding therapeutic interventions, such as the administration of hyperosmolar therapy, surgical decompression, or mechanical ventilation adjustments [6]. Given the critical nature of managing ICP, non-invasive, easily accessible tools like ONSD measurement can provide valuable information in high-risk ICU environments [7,8]. While studies have demonstrated a correlation between ONSD and ICP, the relationship between ONSD and mortality outcomes in ICU patients remains less explored. Investigating whether a larger ONSD is associated with higher mortality rates in critically ill patients could provide important insights into the prognostic value of this measurement. Understanding this correlation may help clinicians to better manage patient care, predict outcomes more accurately, and improve overall ICU management practices.

The primary objective of this present study was to evaluate ONSD as a prognostic tool for mortality outcomes in ICU patients, contributing to the growing body of knowledge on non-invasive methods for assessing critical care parameters. The secondary objective was assessing ONSD changes from Day 1 to Day 10 and correlating ONSD with inflammatory biomarkers like C-reactive protein (CRP) and procalcitonin (PCT).

## Materials and methods

This prospective cohort study was conducted in the Medical ICU of Rashid Hospital, Dubai, UAE, and was designed to examine the relationship between ONSD and mortality outcomes in critically ill ICU patients. The inclusion criteria were patients who were 14 years or older and had been admitted to the ICU. Exclusion criteria were age <14 years or pre-existing conditions such as optic nerve disorders, TBI, or intracerebral hemorrhage (ICH).

The primary outcome of the study was ICU mortality at 30 days, whereas secondary outcomes included overall mortality at 90 days, ICU length of stay, and duration of mechanical ventilation.

ONSD was measured using a standardized transorbital ultrasonography protocol on Day 1 and Day 10 of ICU admission. Clinical and demographic data, including age, sex, comorbidities (e.g., diabetes mellitus (DM) and hypertension (HTN)), injury severity score, Glasgow Coma Scale (GCS), laboratory data (e.g., liver function tests (LFTs), hemoglobin (Hb), creatinine, urea, PCT, CRP), and information on sepsis, stroke (excluding TBI/ICH), and ventilator support were collected for each patient. Descriptive statistics were used to characterize the study population, including the mean and standard deviation of ONSD measurements.

ONSD measurement protocol

We used a 7-12 MHz linear array probe of the Sonohealth D5CL handheld model (Guangzhou SonoHealth Medical Technologies Co., Ltd., Guangzhou, China). The patient's positioning was supine and 30° head-up. We also used thin hypodermic plastic film as a barrier to avoid any eye infections, and hypoallergenic gel was applied to minimize pressure on the globe to prevent artefactual distortion.

The operator ensured that the probe was perpendicular to the orbital axis, and the standardized depth was 3 mm posterior to the retina. The calculation of the mean of three consecutive measurements was recorded. The operator was blinded to patient outcomes. Inter-observer variability was minimized by using trained sonographers following this standardized protocol.

Data were collected over a six-month period (between November 2024 and May 2025). The total number of patients enrolled and consented was 68. The proposed study was authenticated with pre-approval from the ethical committee (Dubai Academic Health Corporation, approval number: 2024002), and appropriate data management protocols were implemented to ensure the integrity and accuracy of the study.

Statistical analysis was performed using IBM SPSS Statistics for Windows, Version 21 (released 2012; IBM Corp., Armonk, New York, United States). Data were entered into an Excel spreadsheet, and descriptive statistics, including mean and standard deviation for quantitative variables and frequency and proportions for qualitative variables, were calculated. Independent sample t-tests were used to compare quantitative parameters between groups based on prognosis, and paired t-tests were used to compare parameters within groups at different time intervals (Day 1 vs. Day 10), with a significance level set at 5%.

## Results

The patients' ages ranged from 25 to 92 years, with a mean age of 58.29 years and a standard deviation of 15.40. Approximately 13.2% of patients were aged between 25 and 40 years, 27.9% were aged 41 and 55 years, 36.8% were between 56 and 70 years, and 22.1% were over 70 years. Among them, 51.5% of the patients were females, while 48.5% were males. In terms of comorbidities, 64.7% of the patients had DM, while 35.3% had no DM. Regarding HTN, 58.8% had HTN, and 41.2% were free of hypertension (Table 1).

**Table 1:** Demographic data and prevalence of comorbidities among the studied population. DM: diabetes mellitus; HTN: hypertension

Demographic data and comorbidities			
	Age Groups	Count	Percent
	25 to 40 yrs.	9	13.2%
	41 to 55 yrs.	19	27.9%
	56 to 70 yrs.	25	36.8%
	> 70 yrs.	15	22.1%
Sex	Male	33	48.5%
	Female	35	51.5%
DM	Yes	44	64.7%
	No	24	35.3%
HTN	Yes	40	58.8%
	No	28	41.2%

The data for ONSD, the side-specific measurements, revealed that, on Day 1, the mean ONSD for the left side was 3.86 ± 0.35 mm, and for the right side, it was 3.90 ± 0.37 mm. On Day 10, the mean ONSD values were 4.09 ± 0.57 mm for the left side and 4.13 ± 0.59 mm for the right side.

Hb levels showed a mean of 11.51 g/dL (SD = 2.45) on Day 1, which decreased to 10.79 ± 1.71 g/dL by Day 10, with a statistically significant mean difference of 1.19 (p = 0.001). CRP levels were significantly reduced from a mean of 172.84 ± 120.95 on Day 1 to 57.60 ± 82.44 on Day 10, with a mean difference of 125.48 (p = 0.001). PCT levels, however, did not show a significant change, with a mean of 1.66 ± 3.20 on Day 1 and 1.10 ± 2.40 on Day 10, with a p-value of 0.382.

Regarding kidney function, creatinine levels showed a slight increase from a mean of 1.62 ± 2.44 on Day 1 to 2.49 ± 4.64 on Day 10, but this change was not statistically significant (p = 0.119). Urea levels significantly increased from a mean of 60.94 ± 55.19 on Day 1 to 106.09 ± 74.86 on Day 10, with a mean difference of -58.19 (p = 0.001). Sodium (Na) levels also significantly increased from a mean of 135.40 ± 6.31 on Day 1 to 140.57 ± 4.98 on Day 10, with a mean difference of -5.74 (p = 0.001) (Table 2).

**Table 2:** Comparison of the ONSD and various laboratory parameters between the time interval (Day 1 and Day 10) using a paired T-test. ONSD: optic nerve sheath diameter; Hb: hemoglobin; CRP: C-reactive protein; PCT: procalcitonin * P-value is statistically significant

Variables	n	Minimum	Maximum	Mean ± SD	Mean diff	T-value	P-value
ONSD left Day 1	68	3.3	5.3	3.86 ± 0.35	-0.226	-5.60	0.001*
ONSD left Day 10	68	3.4	5.7	4.09 ± 0.57			
ONSD right Day 1	68	3.3	5.4	3.90 ± 0.37	-0.226	-5.72	0.001*
ONSD right Day 10	68	3.4	5.7	4.13 ± 0.59			
Hb Day 1	68	6.1	16.4	11.51 ± 2.45	1.19	3.68	0.001*
Hb Day 10	47	6.8	15.0	10.79 ± 1.71			
CRP Day 1	68	1.6	546.8	172.84 ± 120.95	125.48	6.10	0.001*
CRP Day 10	44	0.4	390.5	57.60 ± 82.44			
PCT Day 1	68	0.06	17.16	1.66 ± 3.20	0.525	0.884	0.382
PCT Day 10	42	0.06	14.19	1.10 ± 2.40			
Creatinine Day 1	68	0.3	18.3	1.62 ± 2.44	-1.14	-1.58	0.119
Creatinine Day 10	48	0.2	31.0	2.49 ± 4.64			
Urea Day 1	68	6.0	284.0	60.94 ± 55.19	-58.19	-4.44	0.001*
Urea Day 10	46	17.0	285.0	106.09 ± 74.86			
Sodium Day 1	68	118.0	154.0	135.40 ± 6.31	-5.74	-5.08	0.001*
Sodium Day 10	47	131.0	151.0	140.57 ± 4.98			

The GCS scores ranged from 3.0 to 10.0, with a mean score of 3.13 ± 0.88, indicating a generally low level of consciousness among patients.

Regarding the LFT, on Day 1, 44.1% of the patients had a deranged LFT, and 51.5% had a normal LFT. By Day 10, the distribution had shifted, with 26.5% having a normal LFT and 27.9% having a deranged LFT. In terms of sepsis, a significant majority (91.2%) of the patients had sepsis, whereas only six (8.8%) did not have sepsis. Most patients (94.1%) did not experience a stroke, while 5.9% had a stroke. With respect to prognosis, the results showed that 66.2% of patients were alive, while 33.8% had died by the end of the study. These findings provide an overview of the clinical conditions and outcomes of the study patients, with a notable prevalence of sepsis in the majority of survivors (Table 3).

**Table 3:** Prognosis and clinical outcome among the studied population.

Prognosis and clinical outcomes		Count	Percent
Sepsis	Yes	62	91.2%
	No	6	8.8%
Stroke	Yes	4	5.9%
	No	64	94.1%
Liver deranged (Day 1)	Yes	30	44.1%
	No	35	51.5%
	Not recorded	3	4.4%
Liver deranged (Day 10)	Yes	19	27.9%
	No	18	26.5%
	Not recorded	31	45.6%
ICU outcome	Died	23	33.8%
	Discharged	45	66.2%

On Day 1, the mean ONSD for the left eye in patients who were alive was 3.67 ± 0.23 mm, while those who died had a high mean ONSD of 3.97 ± 0.36 mm. A similar trend was observed for the right eye, where the mean ONSD for the alive group was 3.68 ± 0.26 mm, compared to 4.02 ± 0.37 mm in the deceased group. In both eyes, the differences in mean ONSD between the alive and dead groups were statistically significant, with p-values of 0.001. On Day 10, the trend continued, with the alive group showing a mean ONSD of 3.70 ± 0.38 mm on the left and 3.71 ± 0.40 mm on the right, while the deceased group had a high mean ONSD of 4.29 ± 0.55 mm (left) and 4.35 ± 0.55 mm (right). The differences in mean ONSD on Day 10 were also statistically significant for both sides, with p-values of 0.001 (Table 4).

**Table 4:** Comparison of clinical parameters of the mean ONSD, CRP, and PCT values among survivors and non-survivors on Day 1 and Day 10 using an independent sample T-test. ONSD: optic nerve sheath diameter; CRP: C-reactive protein; PCT: procalcitonin * P-value is statistically significant

	Outcome										
	Death (non-survivors)				Discharge (survivors)				Mean diff	T-value	P-Value
	n	Mean ± SD	Minimum	Maximum	n	Mean ± SD	Minimum	Maximum			
ONSD left Day 1	45	3.97 ± 0.36	3.5	5.3	23	3.67 ± 0.23	3.3	4.4	-0.301	-4.16	0.001*
ONSD right Day 1	45	4.02 ± 0.37	3.5	5.4	23	3.68 ± 0.26	3.3	4.6	-0.332	-5.17	0.001*
ONSD left Day 10	45	4.29 ± 0.55	3.5	5.7	23	3.70 ± 0.38	3.4	5.3	-0.591	-4.31	0.001*
ONSD right Day 10	45	4.35 ± 0.55	3.5	5.7	23	3.71 ± 0.40	3.4	5.4	-0.641	-5.44	0.001*
CRP Day 1	45	169.75 ± 124.09	1.6	546.8	23	178.87 ± 117.03	29.7	417.8	9.11	0.292	0.771
CRP Day 10	22	73.55 ± 103.82	0.4	390.5	22	41.65 ± 51.03	1.6	176.6	-31.89	-1.29	0.203
PCT Day 1	45	1.49 ± 2.94	0.06	13.34	23	2.00 ± 3.70	0.08	17.16	0.513	0.623	0.535
PCT Day 10	20	1.86 ± 3.32	0.06	14.19	22	0.41 ± 0.45	0.06	1.79	-1.45	-2.033	0.049*

For the CRP levels on Day 1, the alive patients had a mean of 178.87 ± 117.03, while those who died had a mean of 169.75 ± 124.09. This difference was not statistically significant (p = 0.771). On Day 10, the CRP levels for the surviving patients decreased to a mean of 41.65 (SD = 51.03), while the deceased group had a mean of 73.55 ± 103.82; however, the difference was not statistically significant (p = 0.203).

PCT levels on Day 1 showed that the surviving patients had a mean of 2.00 ± 3.70, while the dead group had a mean of 1.49 ± 2.94. This difference was not statistically significant (p = 0.535). On Day 10, however, the alive patients showed a significant reduction in PCT levels, with a mean of 0.41 ± 0.45, while the deceased group had a higher mean of 1.86 ± 3.32, with a statistically significant difference (p = 0.049) (Table 4).

## Discussion

The correlation between the ONSD and mortality outcomes in ICU patients is recognized as a significant prognostic indicator. The present study evaluated the utility of ONSD measurements under various critical conditions, particularly in predicting mortality in patients with elevated ICP. ONSD measurement is a non-invasive, bedside tool that can effectively predict ICP and associated mortality outcomes, making it a valuable addition to ICU monitoring protocols [9]. The ability to quickly assess ONSD allows for timely interventions, potentially improving patient outcomes in critical care settings. Elevated ONSD has been linked to poor outcomes in several studies. In the study of Gultekin & Guven, a median ONSD of 5.95 mm was observed in non-survivors compared to 4.15 mm in survivors, indicating a strong correlation with in-hospital mortality [10]. In cases of intracranial hemorrhage, ONSD values above 6.62 mm significantly predicted brain death, with a 25.529-fold increased risk associated with such measurements [11]. Some studies suggest that other factors, such as age and comorbidities, may also significantly influence mortality outcomes, indicating a need for a comprehensive assessment approach in ICU settings [12].

The present study's findings, including low GCS scores and significant changes in Hb and CRP levels, align with the existing literature that shows the prognostic value of ONSD in predicting mortality. The mean GCS score in the current study was low (3.13 ± 0.88), indicating severe impairment, which is consistent with findings that lower GCS scores correlate with higher ONSD and increased mortality risk [1,2]. Studies have shown that an increase in ONSD is associated with a twofold increase in hospital mortality for each millimeter increase [2]. The significant decrease in Hb and CRP levels over ten days suggests a potential recovery pattern, yet the initial high ONSD remains a critical predictor of poor outcomes [1,12]. Elevated CRP levels have been linked to worse outcomes in TBI patients, reinforcing the importance of monitoring these markers alongside ONSD [1]. The increase in urea and Na levels indicates potential renal impairment, which can complicate recovery and is often associated with higher mortality in critically ill patients [13]. The relationship between ONSD and renal function shows the multifaceted nature of TBI outcomes, where ICP may also affect systemic functions [9].

In the present study, on Day 1, 44.1% of the patients had a deranged LFT, and 51.5% had a normal LFT. By Day 10, the distribution had shifted; 26.5% had a normal LFT, and 27.9% had a deranged LFT. The majority of patients had sepsis (91.2%), most did not experience a stroke (94.1%), and 66.2% survived by the end of the study. This high prevalence of sepsis and significant mortality rate aligns with existing literature, further emphasizing a correlation between sepsis, elevated ICP, and poor prognosis. Pansell et al. noted that elevated ONSD is common in severe COVID-19, suggesting a potential mechanism for increased mortality in septic patients [14]. The low incidence of stroke (5.9%) in this study contrasts with the high ONSD values observed in other studies, indicating that while stroke may not be prevalent, other factors, such as sepsis, significantly impact mortality outcomes.

The present study on ONSD as a prognostic tool for mortality in ICU patients revealed significant differences in ONSD measurements between survivors and non-survivors, both on Day 1 and Day 10. This aligns with existing literature, which supports the use of ONSD as a reliable indicator of ICP and mortality risk in critically ill patients. The study results found mean ONSD values of 3.68 mm for alive vs. 4.02 mm for deceased on Day 1 and 3.71 mm vs. 4.35 mm on Day 10, with p-values of 0.001 indicating statistical significance. A study reported a median ONSD of 5.95 mm in non-survivors, significantly higher than 4.15 mm in survivors, emphasizing the predictive value of ONSD in mortality [15]. Banerjee et al. highlighted the utility of ONSD in guiding management decisions in ICU settings, reinforcing its role in prognostication [16]. While ONSD is a promising non-invasive tool, some studies, like that of Breedt et al., found no significant correlation between ONSD and ICP in certain cases, indicating that further research is needed to clarify its utility in diverse clinical scenarios [17]. The combination of ONSD and GCS measurements may provide a more comprehensive assessment of the TBI severity and prognosis. One study, Hwan Kim et al., found that combining ONSD and grey matter-to-white matter ratio cutoff values improved sensitivity in predicting poor neurological outcomes to 92% [18]. This suggests that integrating multiple parameters, including ONSD and GCS, could enhance prognostic accuracy in TBI patients.

The CRP data did not show a statistically significant difference between survivors and non-survivors on either Day 1 or Day 10. This contrasts with ONSD findings, suggesting that ONSD may be a more sensitive predictor of mortality than CRP in this context. However, the lack of statistical significance in the CRP differences could be due to the small sample size or other confounding factors. The initial PCT levels on Day 1 were not significantly different between the alive and dead groups (p = 0.535). This suggests that PCT level at admission may not be a reliable predictor of mortality in ICU patients. However, the significant difference in PCT levels observed on Day 10 (p = 0.049), with a striking reduction in the surviving patients, indicated that the trend in PCT levels over time may be more informative. The reduction in PCT levels in surviving patients compared to the persistently high levels in non-survivors suggests that PCT kinetics could be a valuable prognostic tool [19]. These findings align with previous research showing that serum PCT levels at ICU discharge can predict post-ICU mortality [19]. The present study extends this concept by demonstrating that PCT trends during ICU stay, rather than initial values, may be more indicative of patient outcome.

Previous research has shown that ONSD has a significant association with ICU mortality in severe TBI patients [1]. An ONSD cutoff ≥ 7.3 mm was found to have high sensitivity and specificity for predicting mortality. Similarly, another study, Sekhon et al., found that each 1 mm increase in ONSD was associated with a twofold increase in hospital mortality [2].

Studies have also indicated that elevated ONSD is associated with poor outcomes in various critical conditions, including COVID-19 and non-traumatic brain injuries. Gultekin et al. found that a median ONSD greater than 5 mm significantly correlated with in-hospital mortality in COVID-19 patients [10]. Bhide et al. highlighted that ONSD measurements can effectively predict ICP and, consequently, patient prognosis in non-traumatic neuro-critical cases [20]. While the correlation between ONSD and mortality is compelling, it is essential to consider that not all patients with elevated ONSD experience poor outcomes. Factors such as timely medical intervention and individual patient resilience can influence survival, suggesting a need for a nuanced understanding of ONSD as a prognostic tool in diverse clinical scenarios [21].

Limitations

The study was conducted in a single center with a relatively small sample of patients. Also, the lack of multivariable regression analysis clarifies that ONSD is associated with mortality but not confirmed as an independent predictor. While ONSD is a valuable prognostic indicator, we recommend its use as an adjunctive tool integrated with other clinical parameters (GCS, sepsis status) rather than as a standalone diagnostic for mortality. Future research should focus on a multimodal scoring system that includes variables like ONSD, GCS, PCT, etc. There is a need for future large-scale multicenter studies with multivariable risk-stratification models to validate ONSD as a clinical tool.

## Conclusions

Our study demonstrated the significant prognostic value of ONSD in predicting mortality outcomes for ICU patients. Elevated ONSD is strongly associated with poor outcomes, including higher mortality, particularly in septic patients. The combination of ONSD with other clinical markers, such as GCS scores and inflammatory markers, may enhance the prognostic accuracy. However, further research is needed to understand the multifactorial nature of mortality risk and to refine the use of ONSD as a prognostic tool in critical care.
